# Accuracy of subject-specific prediction of end-systolic time in MRI across a range of RR intervals

**DOI:** 10.1371/journal.pone.0179011

**Published:** 2017-06-09

**Authors:** Christophe Meyer, Jacques Felblinger, Pierre-André Vuissoz, Laurent Bonnemains

**Affiliations:** 1 U947, INSERM, Nancy, France; 2 IADI, University of Loraine, Nancy, France; 3 Clinical Investigation Center (CIC-IT 1433), CHU Nancy, Nancy, France; 4 Department of Cardiac Surgery, CHU Strasbourg, Strasbourg, France; 5 University of Strasbourg, Strasbourg, France; Nanjing Normal University, CHINA

## Abstract

**Background:**

Prediction of End-Systole time is of utmost importance for cardiac MRI to correctly associate acquired k-space lines during reconstruction of cine acquisitions. This prediction is usually based on the patient’s heart rate using Weissler’s formula, which was calibrated by linear regression within a population and cannot account for individual variability.

**Objective:**

We propose an automatic method to build a personalized model that better predicts end-systole.

**Methods:**

A phase contrast sequence was modified to acquire only central k-space line with 6.6ms temporal resolution, in a slice passing through the aorta during 128 heartbeats in 35 subjects. Segmentation of aorta and detection of end of systolic ejection was automatic. Duration of electromechanical systole duration as function of heart rate was determined for each subject separately.

**Results:**

In comparison with the global models, the adapted cardiac model predicted significantly better both echocardiographic end-systolic reference (bias = 0ms vs 17ms, p<0.001) and MRI measurements (bias = 6.8ms vs 17ms). Favorable impact was shown on the cine reconstruction of the 5 subjects with the higher cardiac variability (p = 0.02).

**Conclusions:**

Personalization of cardiac model to the subject is feasible in MRI and reduces the error of prediction of systole.

## Introduction

Prediction of end-systole time during each cardiac cycle is of utmost importance during cardiac Cine-MRI because it directly influences the way k-space lines are combined during reconstruction. This is especially important for high temporal resolution cine acquisitions [1,2] for which the long acquisition time lasts several heart beats undergoing changing heart rate. To associate k-space lines acquired during different cardiac cycles, the most used reconstruction algorithm, named CAPTOR, considers the line’s acquisition time within the cardiac cycle and modifies this time by a bi-phasic projection into a mean cycle (one phase for the systole and the other for the diastole) [3]. Errors concerning end-systole prediction can therefore create k-spaces filled with lines theoretically belonging to another k-space. We recently showed that these mismatches could lead in important errors in tissue velocity measurement [4]. In this previous study, the projection errors concerned especially mid-cycle tissue displacements, such as early diastole filling and reached a mean of 6cm/s. Two third of these errors were due to the use of a biphasic model and the other third was due to the use of Weisler’s formula. Indeed, although this formula is used routinely and worldwide for cine reconstructions, it is based on data acquired more than 45 years ago with a phonocardiograph [5]. In that study, Weissler *et al*. performed a linear regression on the delay between both heart sounds within a cohort of subjects. This formula predicts the duration of systole and of diastole, within a general population. However, the model was not designed to predict variations within different cardiac cycles of a single subject. It cannot cope with physiological differences among subjects due for example to pathological conditions, diurnal variation in the systolic intervals [6], pressure changes [7] or medication that alter systolic or diastolic times [8]. More so, it is known that equations from females and males differ slightly [9,10] and that the left ventricular ejection duration increases independently from heart rate from infancy to puberty [11,12] and is prolonged in the elderly [10,13]. These inter-individual differences should be taken into account.

**Fig 1:**
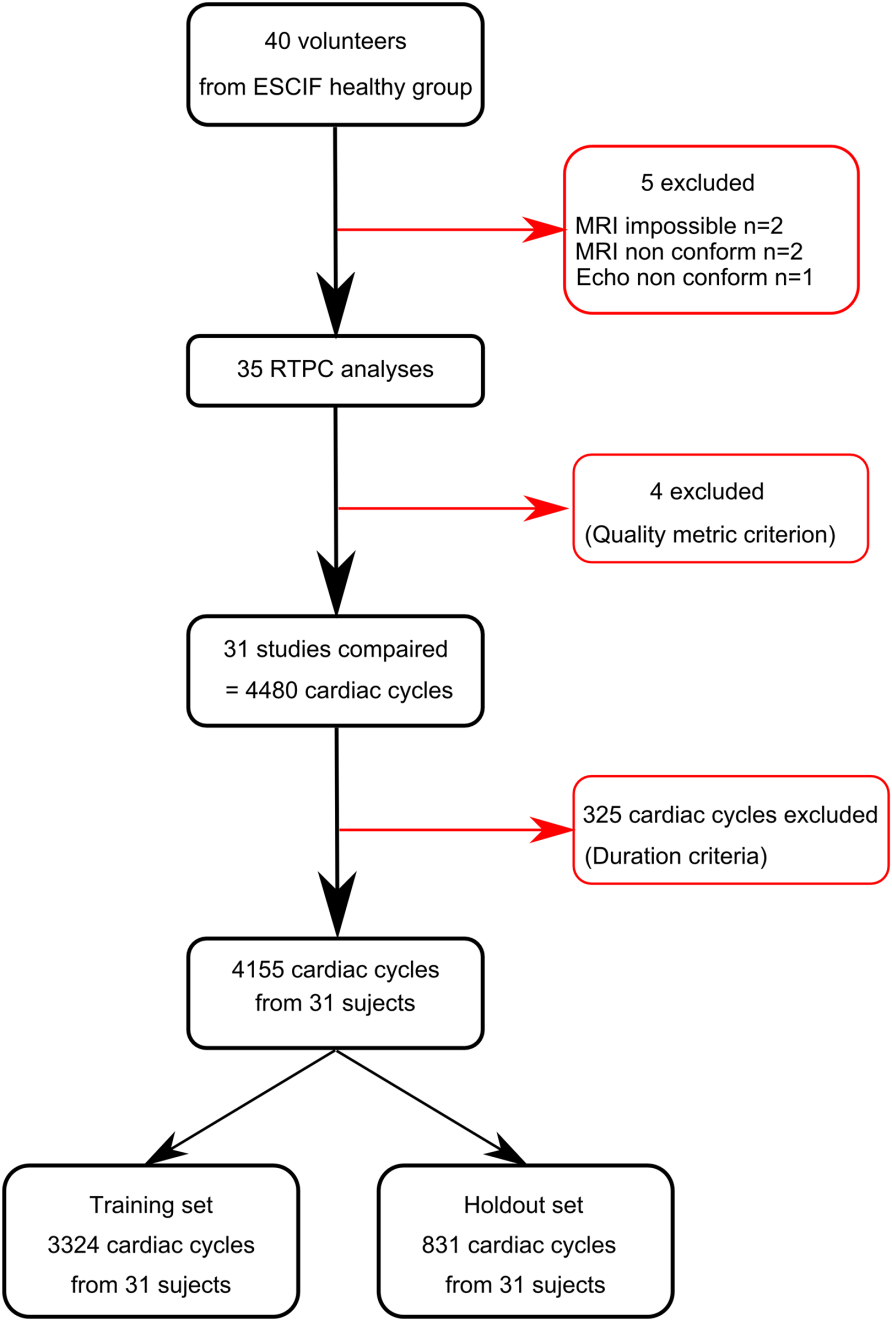
Flow chart of the study.

Our goal in this study was to propose a fully automated method to compute a personalized prediction of end-systole time. The study population comprised 40 subjects. Phase contrast data were acquired at high temporal resolution for 100s and used to automatically compute personalized cardiac models for 35 of them (Flow chart of the method presented in Fig 1). The models were validated against echocardiography and Weisler’s global model. Endly, myocardial velocities after reconstruction using Weisler’s formula and our personalized model were compared for the subjects with the higher heart rate variability.

## Methods

### Study population

The population of this study corresponds to the “healthy” subgroup of the ESCIF project, which is an ancillary study of the Stanislas Cohort [14] (monocentric familial longitudinal cohort comprised of 1006 families from the Nancy region in France). It has been registered in clinical trials under the ID: NCT01391442. Every adult volunteer from the Stanislas Cohort, recruited for the Stanislas fourth visit between 29/03/2013 and 22/11/2013, was considered eligible for the ESCIF program, if the MRI scanner was available. They were proposed to participate in the ESCIF program and those who were included in the “healthy group” of ESCIF constitute the population of our prospective study. These forty consecutive volunteers were qualified as healthy after a complete clinical and echocardiographic examination. The protocol of the ESCIF program is provided in S1 File. All volunteers gave written informed consent. The study complied with the Declaration of Helsinki regarding medical research on human subjects and was approved by the local ethic committee (CPP Est 3 –NANCY, France—mail: cppest.3@chru-nancy.fr).

### Creation of the patient adapted cardiac model

#### MR imaging protocol

**Fig 2:**
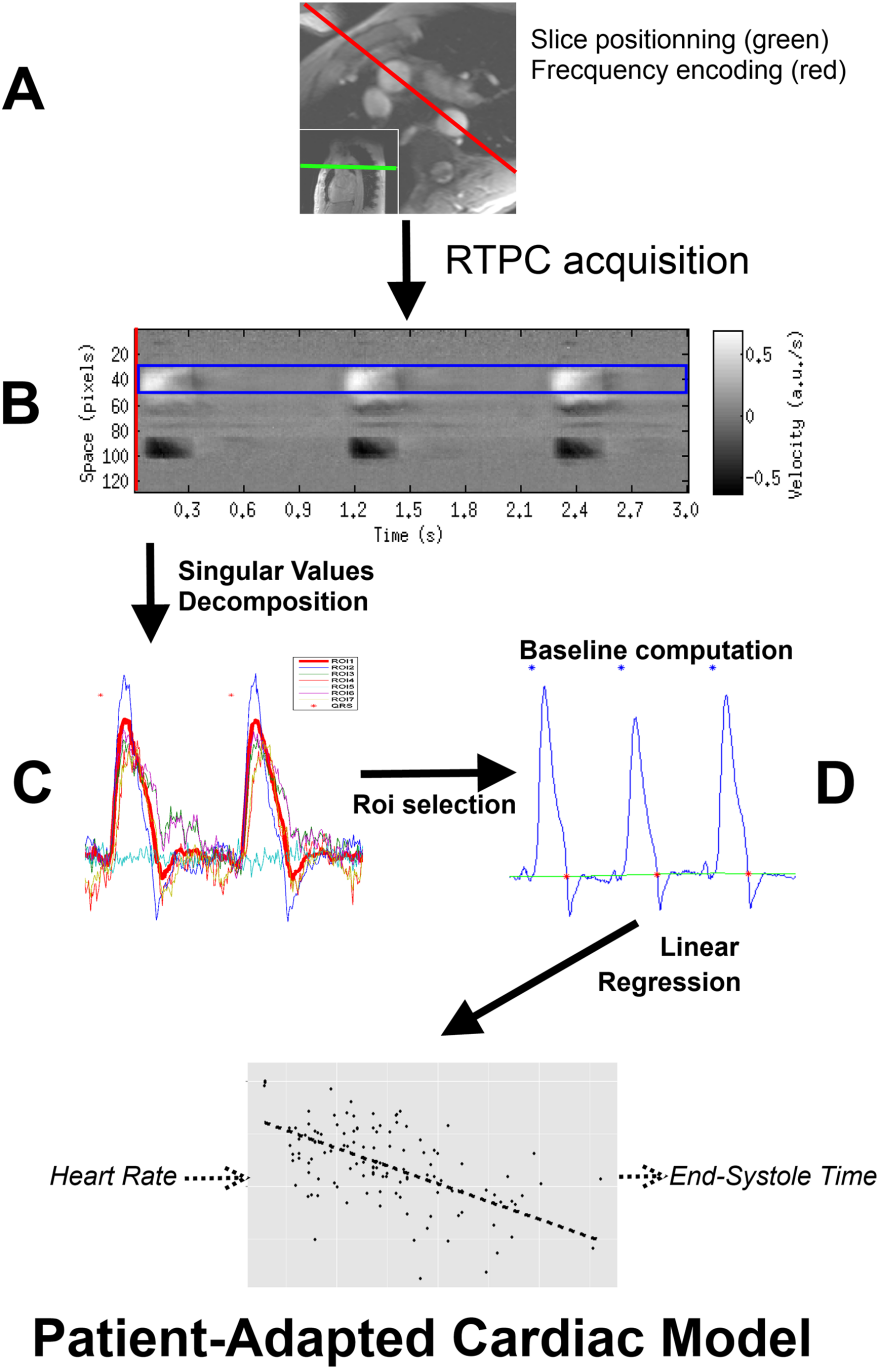
Flow chart of the RTPC method. The acquisition slice is positioned as presented in the bottom left corner of subfigure (A). The red line represents the frequency encoding direction. Subfigure (B) is a 3-cardiac-cycle subset of a RTPC acquisition. The blue rectangle is the result of the automatic segmentation and corresponds to the ascending aorta. Subfigure (C) presents the first step of the automatic segmentation, the singular value decomposition. An automatic ROI selection selects the appropriate curve, represented with a bold red line. Subfigure (D) presents the results of the baseline computation algorithm on this selected curve. The patient adapted cardiac model is then computed by linear regression.

Cardiac MRI studies were performed on a 3T Signa HDxt scanner (General Electric, Waukesha, WI) using an eight-element cardiac phased-array coil with subjects in supine position. First, localizing scans were recorded to determine the orientation of the aorta cross. Second, a standard phase contrast sequence “FastCINE PC” was acquired, twice, in a slice positioned perpendicular to the ascending aorta (normal to the direction of blood flow) and three centimeters above the sinotubular junction to avoid as much as possible the pulmonary artery trunk (Fig 2A). For subjects with normal anatomy, this orientation corresponds roughly to an axial slice. The first acquisition was performed in breath-hold with 6 k-space lines per segment and the second one in free-breathing with 1 k-space line per segment and three excitations. Subsequently, a Real-Time Phase Contrast (RTPC) sequence was acquired during 128 heart beats in free-breathing within the same slice. This RTPC sequence was adapted from the 2D segmented Fast Gradient Recalled Echo “FastCINE PC” (version 15M4), which was modified to acquire only the central k-space line, similarly to the RACE sequence [15,16]. One-directional through-slice interleaved velocity encoding was used with parameters described in Table 1. The orientation of the frequency encoding direction was angulated to be parallel to a line joining the ascending aorta and the descending aorta as illustrated by the red line in Fig 2A. During acquisition, raw data were visualized and recorded using in-house real-time system [17]. ElectroCardioGram (ECG) was recorded and triggering was performed by a custom MagLife patient monitoring system (Schiller, Wissembourg, France) [18]. Data were anonymized before reconstruction. RTPC processing was completely automatic and required no subject-specific parameter tuning.

**Table 1:** MRI scan parameters.

Parameter	RTPC	BHPC	FBPC
Field Of View (mm)	350	320	320
Slice thickness (mm)	8	8	8
Flip angle (°)	15	15	15
Bandwidth (kHz)	62.5	62.5	62.5
Acq. matrix (Freq x Phase)	256x1	256x128	256x128
TR (ms) / TE (ms)	6.6 / 3.4	7.0 / 3.8	7.0 / 3.8
Venc (cm/s)	150	150	150
Excitations repetitions	1	1	3
k-Lines per segment	1	6	1
Temporal resolution (ms)	6.6	42	7.0
Typical scan duration (s)	128	21	378

*RT* real-time, *PC* phase contrast, *FB* free-breathing, *BH* breath-hold

#### Reconstruction of RTPC data

RTPC data were reconstructed offline using Matlab (R2013a, The Mathworks Inc., Natick, MA). Data were 1D-Fourier transformed along the frequency encoding direction into 1D+t image space. Only the central 50% FOV (128 pixels) was kept to reduce noisy air regions and processing time, while checking that the ascending aorta was included. For the same reasons, only two coil elements were used (anterior top right and anterior bottom right).

Velocity was computed by subtraction of 2 consecutive echoes, leading to a temporal resolution of 1 TR using Shared Velocity Encoding [19]. No image-based velocity offset correction technique was used during reconstruction. Final 1D+t velocity map (Fig 2B) was computed as magnitude weighted sum of velocity map of both channels.

#### Automatic spatial segmentation

To automatically segment the aorta, the 1D+t velocity map (Fig 2B) underwent singular value decomposition. The undersampled images corresponding to the three first singular values were further simplified with a threshold filter. The use of thresholding after the resolution of an ill-conditioned inversion problem is well known in the literature [20–23]. The pixels remaining after this thresholding were grouped into contiguous regions of interest (ROI). For each ROI, the time course of the mean velocity in the ROI was computed, as illustrated in Fig 2C. The ROI with the velocity time course having the highest power in the cardiac spectral band (using the subject’s mean heart frequency +/- 0.03 Hz) was found and used to select all ROIs having more than 40% of this maximum power and a positive mean velocity. Among those ROIs, the largest ROI (in pixels) was chosen.

#### Automatic detection of end-systolic time

The resulting velocity time course (Fig 2D) was 128 cardiac cycles long and was interpreted as the consequence of the ascending aortic blood flow [24] because, physiologically, aorta is the location of highest variations of velocity in the chosen slice and is the largest vessel. The absolute values of velocity were not used directly because they derived from the projection of all velocities in the slice along the phase encoding direction (as only the central k-space line was acquired). However, the timings of the systolic velocity features were measured.

Duration of systole was computed by measuring the delay between the ECG trigger (R-wave) and the end of forward systolic velocity:

1. To ensure a correct detection of the R wave, the following algorithm was implemented: Each cardiac cycle was analyzed except when an obvious error in QRS detection on the ECG occurred (i.e. cycles with instantaneous heart rate below 30 or above 120 bpm) or ectopic beats were detected (i.e. cycles with instantaneous heart rate differing by more than 20% both from the median cycle duration and from the previous cycle duration) in which case the cardiac cycle, the one before and the one after were discarded. In order to reduce R-wave detection jitter, the online R-wave detection [25] was post-processed. A QRS pattern was computed for each subject as the mean of a fraction of the ECG signal around the online detection. Detections were repositioned at the location of maximum correlation of the QRS pattern over the ECG (summed over all ECG leads).
2. The velocity curve was further processed for detection of end-systolic time. Systolic waves (called S-waves) were automatically detected on the curve as the peaks with the maximum projected velocity per cardiac cycle. The time of end of forward systolic velocity was recorded, defined by the S-wave intersecting the baseline (Fig 2D). The baseline was computed in 10 iterations as follows: a 0.5 Hz low-pass filtered version of the velocity curve generated an initial baseline, then, using histogram analysis, extreme values of the velocity curve were set to baseline and the threshold for extreme values was lowered, at each iteration, by one discrete interval at both sides of the histogram.

When the estimated systole duration was not of physiological value for our population (below 100 ms or above 500 ms) the cardiac cycle was discarded.

#### Patient-adapted cardiac model

To construct each personalized, patient-adapted cardiac model (PACM), a training dataset composed of the first 80% cardiac cycles was constituted. The model was deduced after a robust linear regression between systolic time measured by RTPC and heart rate. A goodness-of-fit quality metric was used to automatically detect and exclude subjects for whom the PACM linear regression was of bad quality. The quality metric was the standard deviation of the residuals of the regression (named SD in tables Table 2 and S1 Table). Since we targeted high temporal resolution, a value of the metric in excess of 10 ms for the PACM was considered indicative of bad quality, in which case the subject was excluded from further analysis.

**Table 2:** Slope and intercept of global cardiac models (linear regression of intervals over heart rate).

Model	Sex	Slope [s/bpm] (CI)	Intercept [s] (CI)	SD [s]	R^2^
Weissler	M	-0.0018 (a)	+0.456 (a)	0.014	(a)
Weissler	F	-0.0016 (a)	+0.461 (a)	0.014	(a)
PACM averaged	M	-0.0016 (+/- 0.0001)	+0.441 (+/- 0.007)	0.023	0.96
PACM averaged	F	-0.0018 (+/- 0.0001)	+0.464 (+/- 0.007)	0.016	0.96
Echography		-0.0020 (+/- 0.0002)	+0.488 (+/- 0.002)	0.017	0.54

All regression coefficients are significant to P < 0.005

a = data not available

SD = standard deviation of the residuals of the regression

CI = confidence interval at 95% level

R^2^ = determination coefficient of the regression systolic time = f(heart rate)

### Validation of PACM

#### Doppler echocardiography protocol

All volunteers underwent echocardiographic examination within two hours before the MRI scan. The examination was performed using a Vivid 7 Ultrasound system (General Electric Vingmed Ultrasound, Horten, Norway) by an experienced cardiologist, blinded to the MRI results. The volunteers were in a quiet state without sedation. Tissue Doppler Imaging (TDI) of the four-chamber (horizontal long axis) view was acquired with electrocardiogram recording. The Doppler sample volume was positioned at 1 cm of the septal insertion site of the mitral valve leaflets and adjusted as necessary to cover the longitudinal excursion of the annulus in both systole and diastole. Velocity curve was recorded for at least three consecutive cardiac cycles. The temporal resolution of the TDI scan is not directly accessible on our ultrasound system but is to be 200 frames/s according to literature with the same hardware [1,4,26] and to the user guide.

Data were anonymized and analyzed offline by the same cardiologist blinded to all volunteer data. Offline post-processing was performed with the EchoPAC software (EchoPAC PC SW-Only V6.1.0, General Electric Vingmed Ultrasound, Horten, Norway).

For each cardiac cycle, the following data were recorded: 1/ systole duration defined as the delay from start of (isovolumetric) contraction to start of (isovolumetric) relaxation; 2/ duration of the cardiac cycle (duration between the beginning of two contractions).

#### Comparisons of models

Three kinds of comparisons were performed:

1. The linear regression over the entire population, hereafter called “PACM averaged”, was compared to the original S1S2 model from Weissler *et al* [9]. The differences between both models were computed in the population of the study.
2. PACM, PACM averaged and Weissler’s model were compared in terms of prediction of end-systole time to echocardiographic data using a Bland and Altman analysis. Biases were compared with student tests.
3. PACM, PACM averaged and Weissler’s model were compared in terms of prediction of end-systole time performed on the last 20% cardiac cycles (holdout set) of each volunteer using Bland and Altman analyses. Biases were compared using student tests.

### Illustration of potential impact on cine MRI

To illustrate the potential temporal smearing in the reconstruction induced by the models [4], the five subjects with highest heart rate variability were selected. The size of the sample (5 subjects) was chosen because we identified 5 subjects with RR standard deviations higher than 60ms. This threshold (60ms) was already used in a previous study [4]. Cine free breathing phase contrast raw data were reconstructed using the established CAPTOR [3] cine reconstruction algorithm and using PACM. The peak velocity of the early diastole filling (E wave) were recorded and compared with a paired one-sided Wilcoxon sum rank test (R function: wilcox.test).

### Statistics

Data were expressed as mean ± standard deviation [minimum value; maximum value]. Statistical analysis was performed using R 3.0.3 (R Foundation for Statistical Computing, Vienna, Austria) [27]. A P-value of less than 0.05 was considered statistically significant. This study complies with the Standards for Reporting Diagnostic accuracy studies (STARD statement), as illustrated by S2 File.

## Results

**Fig 3:**
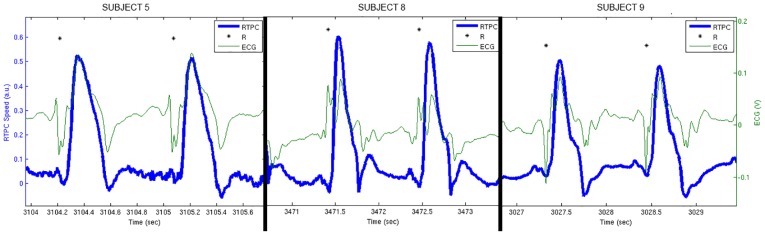
Three examples of small portion of RTPC signal (thick blue line) displayed along with superimposed ECG (green line) for illustration of simultaneous systolic ejection of blood and magneto-hydrodynamic effect. The subjects are respectively subjects 5, 8 and 9. The black stars represent the R wave detection.

The Flow Chart of the study is presented in Fig 1. Forty volunteers were initially recruited. Two volunteers were discarded because MRI was not performed (claustrophobia). Two volunteers were discarded because the MRI acquisitions were not conform (one technical failure and one error from the radiographer). One study was discarded because the echocardiography was not complete. Therefore, thirty-five subjects compose the population of the study (age 52±11 years, 19 females). No adverse events were reported during the study. Four studies were excluded (89% success rate) under the quality metric criterion (standard deviation of the residuals of the regression > 10ms), resulting in 31 subjects analyzed in statistical tests. A total of 4480 cardiac cycles were processed in RTPC. The instantaneous heart rates during these 4480 cardiac cycles were: 67±9.5 bpm [minimum = 44; maximum = 114]. 325 cardiac cycles were automatically discarded under the described acceptance criteria (cardiac cycle length out of bounds). Three examples of accepted RTPC signals displayed along with superimposed ECG are presented in Fig 3 for illustration of simultaneous systolic ejection of blood and magneto-hydrodynamic effect. Finally, the analysis of models was performed on 4155 cardiac cycles: 3324 (training set) for the calibration of PACM and global models and 831 (holdout set) for the validation.

**Fig 4:**
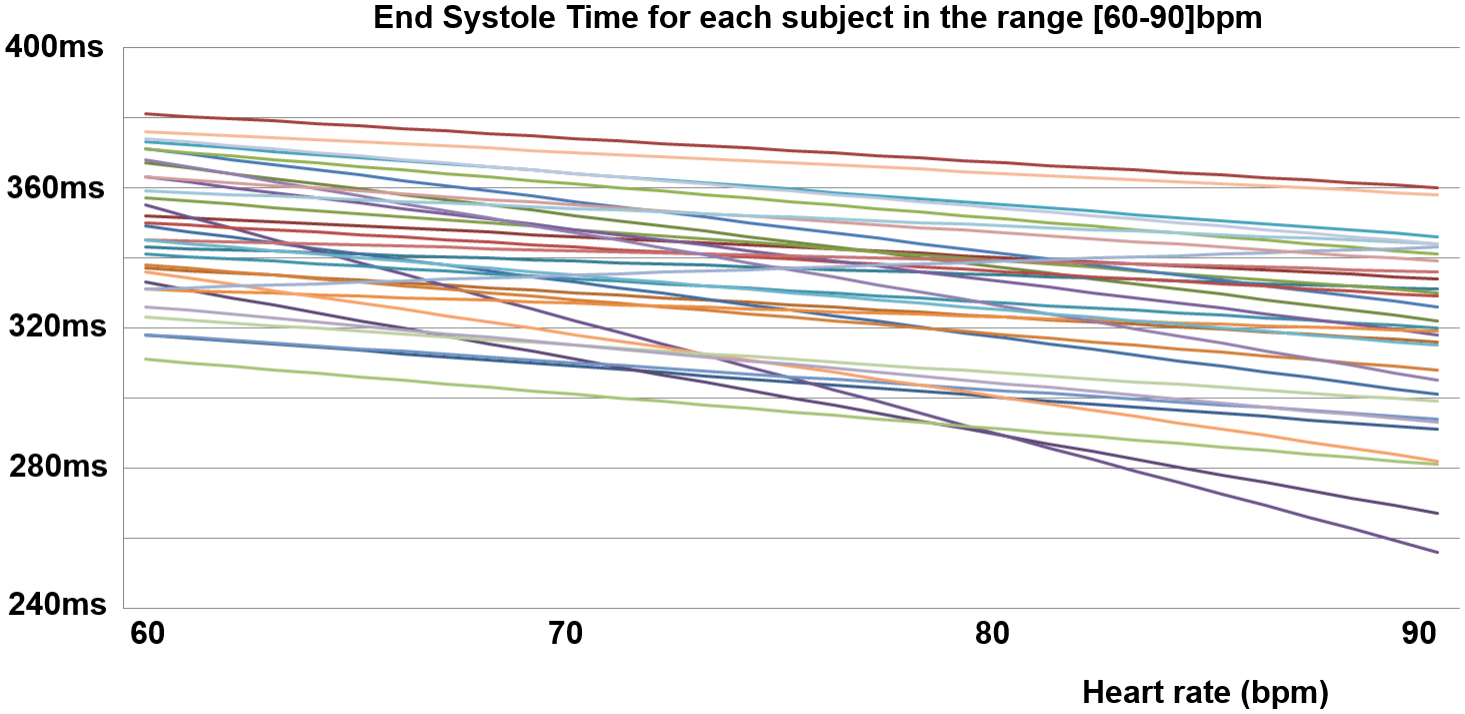
Presentation of the 31 patient-adapted cardiac models. For most subjects, the main difference consists in an offset (most lines are parallels) but for a few subjects there is a major difference in the slope of the model.

For each subject, slope and intercept of the PACM are presented in S1 Table and the corresponding regression lines are illustrated in Fig 4.

The linear regression over our entire population (PACM averaged) is presented in Table 2 along with Weissler’s model [9] in normal individuals. The limits of agreement between the two global models (Weisler—PACM averaged), within the 4480 cardiac cycles, were 4.4 ms for males (maximum error = -7.8 ms) and 2.8 ms for females (maximum error = +16 ms). The bias between the two methods were 2.0±0.1 ms for males and 10.7±0.1 ms for females.

**Fig 5:**
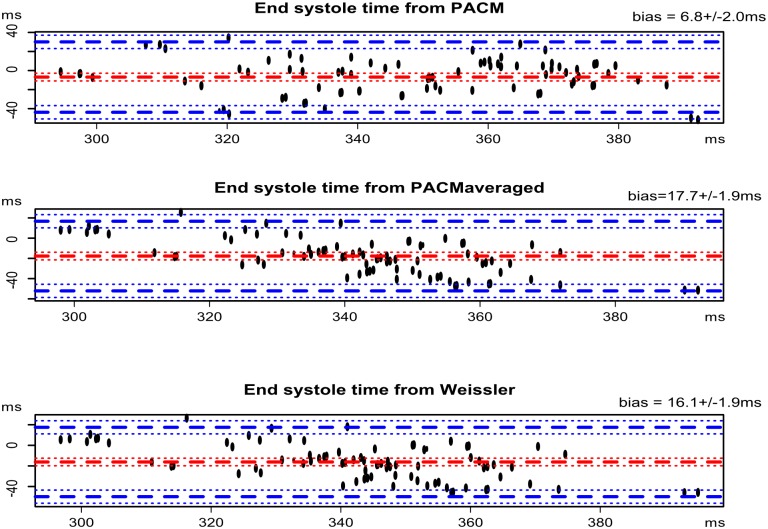
Three Bland and Altman diagrams comparing the reference echocardiography (94 cardiac cycles) with respectively (A) the patient-adapted cardiac model, (B) the PACM-averaged, and (C) Weissler’s model.

**Fig 6:**
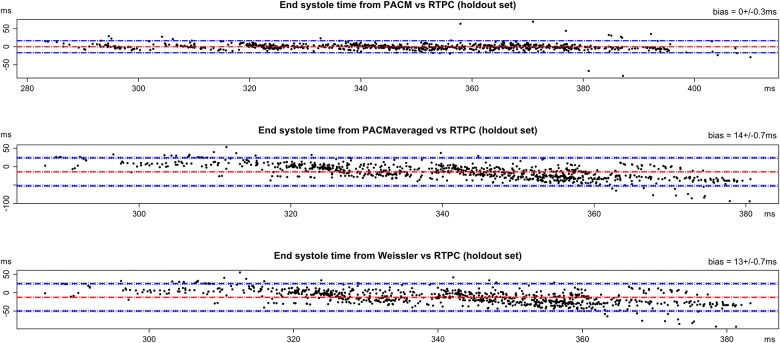
Three Bland and Altman diagrams comparing end-systole time predicted with RTPC in the holdout set (831 cardiac cycles) with respectively (A) the patient-adapted cardiac model, (B) the PACM-averaged, and (C) Weissler’s model. Eight points (8 out of 831 <1%) were discarded from the analysis: in two cases (same subject), RR duration were abnormally long without modification of the systole time (this could be explained by non-conducted P wave for example), whereas in 6 cases (same other subject), end-systole time was abnormally longer than the predictions based on RR duration this could be explained by extra-systole for example).

The echography analysis was performed on 94 cardiac cycles, corresponding to 31 patients. The instantaneous heart rates during these 94 cardiac cycles were: 67±9.5 bpm [minimum = 47; maximum = 95]. The end-systole times measured by echocardiography were 368±33 ms [minimum = 283; maximum = 470]. The end-systole times measured by RTPC were 340±28 ms [minimum = 138; maximum = 565]. The Bland and Altman diagrams comparing the 3 models with the reference echocardiography are presented in Fig 5. The bias were respectively 16.1±1.9 ms for Weisler’s model, 17.7±1.9 ms for PACM averaged and 6.8±2.0 ms for PACM. The three bias were different from zero (p<0.01) and PACM bias was different from the two others (p<0.008). The Bland and Altman diagrams comparing the 3 models with the end-systole time assessed by RTPC in the holdout set are presented in Fig 6. The bias were respectively 13±0.7 ms for Weisler’s model, 14±0.7 ms for PACM averaged and 0±0.3 ms for PACM. The two global models bias were different from zero (p<0.01) and PACM bias was different from the two others (p<0.008).

The five subjects with the higher RR variation were subjects 5, 7, 9, 17, 21. The mean RR standard deviation for these subjects was 74ms (minimum = 62ms, maximum = 89ms). The reconstruction of the tissue phase mapping acquisitions of these five subjects using PACM and Weissler’s method revealed significant differences concerning the E wave peak value (p = 0.02), with higher E peaks for PACM driven reconstruction.

## Discussion

We developed a method to obtain automatic measurements of end-systole times in MRI with very high temporal resolution. This allowed the creation of a subject-adapted cardiac model able to predict the end-systole time. The predictions of end-systole with this automatic and personalized model were better than the usual prediction model proposed by Weissler *et al*. When averaged in our whole population, our results were consistent with Weissler’s model [9]. Indeed, the limits of agreement between the PACM averaged (obtained from simple averaging) and Weissler’s model was 2 ms in our male population. When PACM and Weissler’s model were compared to echocardiographic data, PACM lowered the limit of agreement of prediction of systolic time with a ratio close to 2.5 (16-17ms vs 6.8ms). This ratio was higher between PACM and Weissler’s model limits of agreement when the two models were compared to RTPC measurements performed within the holdout set. This supports the idea that a personalized, patient-adapted cardiac model is of importance in order to accurately estimate the systole position in the cardiac cycle, which in turn is essential to reconstruct cine MRI with high temporal resolution. For temporal resolution used in clinical cardiac MRI, typically 30 ms, it seems that the small temporal errors induced by Weissler’s model have low or no impact on the image quality. Nevertheless, as shown in the five examples selected with RR standard deviation above 60ms, it should not be neglected when RR interval variability is high. The consequences on velocity measurements of small temporal misalignments in acquisition collected in k-space over several heart beats have already been described [4]. The effects within the subset of 5 subjects with higher RR variability were therefore fully expected. A better prediction of systole time seems particularly attractive for high temporal resolution cine sequences or sequences triggered on an acquisition window.

While reduced with PACM, errors in prediction of systole when compared to echocardiography were rather high with a bias close to 7ms. This bias may be explained the fact that we did not use the same definition of systole for both modality. With RTPC, we measured the delay between R-wave on ECG and end of forward blood ejection in the aorta, which defines electromechanical systole. With echocardiography, we measured the delay between cardiac isovolumetric contraction and the end of S-wave, which corresponds to mechanical systole. This bias is also influenced by the jitter in R wave detection during MRI. ECG detection of the R-wave in MRI environment is known to be difficult (especially at high fields such as in our case) and comes with some jitter, which gets reflected in the computation of the end-systolic time [28]. We tried to minimize this problem by repositioning the detection of the QRS complex in an automatic post-processing step based on pattern matching.

Our automatic method was successfully applied in only 31 of 35 subjects. An automatic quality metric was used to detect subjects in which the method failed. In a clinical implementation, this knowledge could be used to re-plane the scan and reacquire the data or to fall back to using Weissler’s model. In the present study, we simply excluded the subjects. This quality metric was not based on the usual r^2^ goodness-of-fit metric because we expected some subjects to have almost constant systole duration in which case the r^2^ would be very low (due to zero variation of the outcome variable: systole duration) despite the (constant) model being of good quality. Causes of failure were the presence of vessels (superior vena cava, pulmonary artery trunk, other vessels) in the scan plane aligned with the ascending aorta in the projection axis (phase encoding axis) resulting in a mix of flow patterns in the same ROI. This could be avoided by performing a scout scan in the same plane as RTPC beforehand, although it would prolong the total scan duration. In fact in our implementation, a complete k-space was acquired before RTPC as a pre-scan in order to get an image (data not exploited in this article). Even if it did not occur in our population, it is conceivable that the automatic ROI selection could fail and select another vessel than the ascending aorta altogether. This problem could be avoided using a more robust automatic criterion or a manual spatial segmentation (at the expense of losing the fully automatic feature of the presented technique). Other difficulties were (i) aliasing in the velocity signal that could not be correctly unwrapped (an unwrapping algorithm might be used [29]) and (ii) velocity noise at the boundary of the lung (mildly seen in Fig 1 but in that case it caused no problem).

With the setting of our study, the additional scan time would be around 100s (80% of 128 cardiac cycles at 60 bpm). However, this additional time could probably be optimized. Indeed, the time needed depends on the heart rate variations of the subject. If such variations are important but rare, the learning set must be large. If the variations are frequent (for example in case of respiratory arrhythmia in children), the learning set could be reduced drastically. The impact of the proposed reconstruction method on other cine MRI acquisitions such as SSFP 2D images was not investigated in this study. Of course, the impact of a personal model will be all the more significant when the behavior of the heart will be atypical (for example, when the subject presents arrhythmia). The improvement in the 2D images would be null for a subject exactly complying with Weissler formula (which is a mean model). In a previous study [4], we assessed the temporal misplacement of k-space lines due to the use of Weissler formula during the reconstruction of MRI data acquired in a children population (children present respiratory arrhythmia). The width of the Gaussian curve representing the temporal misplacements was ±10ms and reached 20ms in early diastole during the isovolumic relaxation. Therefore, we cannot expect any effects on 2D images with the actual usual temporal resolution (typically 30 images/cycle, ie 35ms). The spatial resolution of the image is also crucial. However, current technical improvements (such as increase in B0 field) will probably open the door to new image resolution (temporal and spatial) in the next decade. We can expect improvements in the quality of 2D images for small structures moving in early-diastole, such as auriculo-ventricular valves acquired with high temporal and spatial resolution. In the meantime, for current clinical use, Weissler formula is sufficient to reconstruct actual 2D morphologic images.

Another limitation of the proposed method was that the RTPC MRI acquisitions were performed in free-breathing, whereas the ROI was kept fixed. However, the antero-posterior displacement of the aorta due to breathing in axial slices is very small [30]. Further development could include motion compensation and ROI tracking in time. We did not use parallel imaging for our RTPC acquisition because only the central k-space line was acquired, and thus parallel imaging could not reduce the number of k-space lines further. We nevertheless made use of the local sensitivity of the multi-channel array coil by using only 2 elements, which gave the most signal from the ascending aorta, therefore reducing contamination of velocity by noise coming from other channels. Furthermore, this study was based on classical segmented cine imaging with Cartesian k-space trajectories. Its conclusions cannot be transposed to new complex acquisition strategies which do not use Feinstein’s model, such as reconstruction with low resolution images for cardiac phase recognition or k-t based reconstructions (which have specific temporal frequency underlying hypothesis) [4].

## Conclusion

This study showed that the subject specific end-systolic time is better predicted by a linear model whose parameters are adapted to the subject rather than by fixed global model parameters. A fully automatic method to adapt the parameters of a linear cardiac model to the subject was proposed and validated against echocardiography. Personalization of cardiac model to the subject is feasible in MRI and reduces the error of prediction of systole. It shows promise in reconstructing high quality high temporal resolution phase contrast cine acquisitions. This personalized model requires a specific MRI acquisition that adds some complexity to the scan protocol. This is certainly useful when high temporal resolution is needed in patients with high RR variability. In other cases, the usual Weisler’s formula may be sufficient.

The principles of the method we proposed could be generalized to a radial or spiral acquisition with high temporal resolution where the information of the central k-space point could be used to auto-calibrate the model used during reconstruction. However, this exciting perspective requires further investigations.

## Supporting information

S1 File Protocol of the ESCIF program. Initial version in French and translation in English.
(PDF)

S2 File STARD checklist. (DOCX)

S1 Table Sex, age, slope, intercept and quality metric of the patient-adapted cardiac model for each subject. Discarded subjects (under the quality criterion) are marked with an ‘x’.
(DOCX)

## References

[1] Meyer C, Bonnemains L, Marçon F, Marie P-Y, Felblinger J, Vuissoz P-A. Longitudinal myocardial peak velocities using high temporal resolution phase-contrast and simple averaging are comparable to tissue Doppler echocardiography. MAGMA 2014;27:211–8. 10.1007/s10334-013-0405-4 24013857

[2] Jung B, Föll D, Böttler P, Petersen S, Hennig J, Markl M. Detailed analysis of myocardial motion in volunteers and patients using high-temporal-resolution MR tissue phase mapping. Journal of Magnetic Resonance Imaging 2006;24:1033–1039. 10.1002/jmri.20703 16947325

[3] Feinstein JA, Epstein FH, Arai AE, Foo TKF, Hartley MR, Balaban RS, et al Using cardiac phase to order reconstruction (CAPTOR): A method to improve diastolic images. Journal of Magnetic Resonance Imaging 1997;7:794–798. 10.1002/jmri.1880070505 9307903

[4] Bonnemains L, Odille F, Meyer C, Hossu G, Felblinger J, Vuissoz P-A. Is High Temporal Resolution Achievable for Paediatric Cardiac Acquisitions during Several Heart Beats? Illustration with Cardiac Phase Contrast Cine-MRI. PLoS ONE 2015;10:e0143744 10.1371/journal.pone.0143744 26599755 PMC4658039

[5] Weissler AM, Harris WS, Schoenfeld CD. Systolic time intervals in heart failure in man. Circulation 1968;37:149–59. 5640345 10.1161/01.cir.37.2.149

[6] Weissler AM, Kamen AR, Bornstein RS, Schoenfeld CD, Cohen S. The effect of deslanoside on the duration of the phases of ventricular systole in man. Am J Cardiol 1965;15:153–61. 14254808 10.1016/0002-9149(65)90449-2

[7] Flessas AP, Kumar S, Spodick DH. Effects of the Valsalva maneuver on the cardiac systolic intervals: Beat-to-beat versus timed analysis. American Heart Journal 1970;80:522–31. 10.1016/0002-8703(70)90201-2 5471214

[8] Boudoulas H, Rittgers SE, Lewis RP, Leier CV, Weissler AM. Changes in diastolic time with various pharmacologic agents: implication for myocardial perfusion. Circulation 1979;60:164–9. 10.1161/01.CIR.60.1.164 376175

[9] Weissler AM, Harris WS, Schoenfeld CD. Systolic Time Intervals in Heart Failure in Man. Circulation 1968;37:149–59. 10.1161/01.CIR.37.2.149 5640345

[10] Willems JL, Roelandt J, Geest HD, Kesteloot H, Joossens JV. The Left Ventricular Ejection Time in Elderly Subjects. Circulation 1970;42:37–42. 10.1161/01.CIR.42.1.37 5425593

[11] Golde D, Burstin L. Systolic Phases of the Cardiac Cycle in Children. Circulation 1970;42:1029–36. 10.1161/01.CIR.42.6.1029 5492536

[12] Harris LC, Weissler AM, Manske AO, Danford BH, White GD, Hammill WA. Duration of the phases of mechanical systole in infants and children. The American Journal of Cardiology 1964;14:448–55. 10.1016/0002-9149(64)90028-1 14215055

[13] Slodki SJ, Hussain AT, Luisada AA. The Q-II interval. 3. A study of the second heart sound in old age. J Am Geriatr Soc 1969;17:673–9. 5788110 10.1111/j.1532-5415.1969.tb02321.x

[14] Siest G, Visvikis S, Herbeth B, Gueguen R, Vincent-Viry M, Sass C, et al Objectives, Design and Recruitment of a Familial and Longitudinal Cohort for Studying Gene-Environment Interactions in the Field of Cardiovascular Risk: The Stanislas Cohort. Clinical Chemistry and Laboratory Medicine 1998;36 10.1515/CCLM.1998.007 9594084

[15] Bock M, Schad LR, Müller E, Lorenz WJ. Pulsewave velocity measurement using a new real-time MR-method. Magnetic Resonance Imaging 1995;13:21–9. 10.1016/0730-725X(94)00077-G 7898277

[16] Möller HE, Klocke HK, Bongartz GM, Peters PE. MR flow quantification using RACE: clinical application to the carotid arteries. J Magn Reson Imaging 1996;6:503–12. 8724417 10.1002/jmri.1880060314

[17] Meyer C, Vuissoz P-A, Felblinger J. Images Preview with a Dynamic Acquisition Real-time System (DARTS). Magnetic Resonance Materials in Physics, Biology and Medicine 2012;25:339–628.

[18] Odille F, Pasquier C, Abächerli R, Vuissoz P-A, Zientara GP, Felblinger J. Noise cancellation signal processing method and computer system for improved real-time electrocardiogram artifact correction during MRI data acquisition. IEEE Trans Biomed Eng 2007;54:630–40. 10.1109/TBME.2006.889174 17405370

[19] Lin H-Y, Bender JA, Ding Y, Chung Y-C, Hinton AM, Pennell ML, et al Shared Velocity Encoding (SVE): A method to improve the temporal resolution of phase contrast velocity measurements. Magn Reson Med 2012;68:703–10. 10.1002/mrm.23273 22139889 PMC3339280

[20] Zhang Y, Peterson BS, Ji G, Dong Z. Energy Preserved Sampling for Compressed Sensing MRI. Computational and Mathematical Methods in Medicine 2014;2014 10.1155/2014/546814 24971155 PMC4058219

[21] Zhang Y, Wu L, Peterson B, Dong Z. A Two-Level Iterative Reconstruction Method for Compressed Sensing MRI. Journal of Electromagnetic Waves and Applications 2011;25:1081–91. 10.1163/156939311795762024

[22] Zhang Y, Dong Z, Phillips P, Wang S, Ji G, Yang J. Exponential Wavelet Iterative Shrinkage Thresholding Algorithm for compressed sensing magnetic resonance imaging. Information Sciences 2015;322:115–32. 10.1016/j.ins.2015.06.017

[23] Zhang Y, Wang S, Ji G, Dong Z. Exponential wavelet iterative shrinkage thresholding algorithm with random shift for compressed sensing magnetic resonance imaging. IEEJ Trans Elec Electron Eng 2015;10:116–7. 10.1002/tee.22059

[24] Langham MC, Li C, Magland JF, Wehrli FW. Nontriggered MRI quantification of aortic pulse-wave velocity. Magn Reson Med 2011;65:750–5. 10.1002/mrm.22651 20882637 PMC3015003

[25] Odille F, Pasquier C, Abaecherli R, Vuissoz P-A, Felblinger J. Signal Analyzer and Event Controller (SAEC) for improved Patient Monitoring and Optimum Synchronization of MR Acquisitions. Proceedings of the 22th Annual Meeting of ESMRMB, Basel, Switzerland: 2005.

[26] Sengupta PP, Khandheria BK, Korinek J, Jahangir A, Yoshifuku S, Milosevic I, et al Left Ventricular Isovolumic Flow Sequence During Sinus and Paced RhythmsNew Insights From Use of High-Resolution Doppler and Ultrasonic Digital Particle Imaging Velocimetry. J Am Coll Cardiol 2007;49:899–908. 10.1016/j.jacc.2006.07.075 17320749

[27] R Development Core Team. R: A Language and Environment for Statistical Computing. Vienna: R Foundation for Statistical Computing; 2009.

[28] Oster J, Pietquin O, Kraemer M, Felblinger J. Nonlinear bayesian filtering for denoising of electrocardiograms acquired in a magnetic resonance environment. IEEE Trans Biomed Eng 2010;57:1628–38. 10.1109/TBME.2010.2046324 20483691

[29] Zhou K, Zaitsev M, Bao S. Reliable two-dimensional phase unwrapping method using region growing and local linear estimation. Magn Reson Med 2009;62:1085–90. 10.1002/mrm.22074 19572389

[30] Meyer C, Vuissoz P-A, Mandry D, Felblinger J. First attempt to motion corrected flow encoding using free-breathing phase-contrast CINE MRI. Journal of Cardiovascular Magnetic Resonance 2012;14:W53 10.1186/1532-429X-14-S1-W53

