# Supplementary material for: Accuracy of subject-specific prediction of end-systolic time in MRI across a range of RR intervals
Source: PLoS One. 2017 Jun 9;12(6):e0179011. doi: 10.1371/journal.pone.0179011 (PMC5466307; doi:10.1371/journal.pone.0179011)
Supplement: S1 File — Initial version in French and translation in English. (PDF) [file pone.0179011.s001.pdf]

Centre hospitalier universitaire de Nancy - Direction de la Recherche et de l'Innovation
**Protocole de Recherche Clinique HPS**<br>**avec DM associé**<br>**Amendement au protocole STANISLAS pour ajout de l' étude ancillaire ESCIF**
protocole principal : HÉRITABILITÉ FAMILIALE, INTERACTIONS GÈNE-GÈNE ET GÈNE-ENVIRONNEMENT DANS LE DOMAINE DES MALADIES CARDIOVASCULAIRES<br>Quatrième visite (à 17 ans) de la Cohorte STANISLAS<br><br>Etude ancillaire : ESCIF

Avis favorable du CPP Est III le 03/02/2011

Autorisation de l'AFSSAPS le 28/01/2011

Version N°3 du 03/08/2012

N°ID-RCB : 2010-A01469-30

Promoteur :

Centre Hospitalier Universitaire de Nancy
Avenue du Maréchal de Lattre de Tassigny
54035 NANCY Cedex

Investigateur principal/coordonnateur :

Nom : Pr. Faiez ZANNAD
Adresse : Centre d'investigation Clinique, Institut lorrain du Cœur et des Vaisseaux Louis Matthieu, 4 rue du Morvan, 54500 Vandoeuvre lès Nancy
Tél : 03 83 15 73 20
Mail : f.zannad@chu-nancy.fr

Responsable de la recherche agissant pour le compte du promoteur / et autorisé à signer le protocole et ses modifications éventuelles au nom du promoteur :

Direction de la Recherche et de l'Innovation
M. Olivier DE PESQUIDOUX – Directeur de la Recherche et de l'Innovation
Hôpital Saint Julien – Rue Foller
CO N°60034
54035 NANCY Cedex

SOMMAIRE

1 Résumé du protocole 3

2 Justification scientifique et description générale de la recherche : 4

3 Objectifs de la recherche : 4

3.1 Objectif principal : 5

3.2 Objectif secondaire : 5

3.3 Critères d'évaluation principaux : 5

3.4 Critères d'évaluation secondaires : 5

4 Conception de la recherche 5

4.1 Modalités de recrutement 5

4.2 Déroulement de l'examen IRM cardiaque 6

4.3 Calendrier de l'étude principale 7

4.4 Identification de toutes les données à recueillir 7

5 POPULATION DE L'ETUDE ANCILLAIRE 7

5.1 Critères d'inclusion 8

5.2 Critères de non-inclusion 8

5.3 Procédure d'arrêt prématuré de la recherche ou d'exclusion POUR UNE PERSONNE DE LA RECHERCHE ET PROCEDURE DE SUIVI DE LA PERSONNE 8

6 Conservation et accès aux données cliniques et PARACLINiques 9

7 Evaluation de la sécurité : 9

8 Statistiques 10

8.1 Description des méthodes statistiques prévues 10

8.2 Nombre prévu de personnes à inclure dans la recherche, avec sa justification statistique 10

1 RESUME DU PROTOCOLE

Contexte :

La Cohorte STANISLAS est une cohorte monocentrique familiale longitudinale de 1006 familles (4295 sujets) de la région de Nancy recrutées en 1993-1995 au Centre de Médecine Préventive (CMP) de Vandoeuvre lès Nancy à l'occasion d'un examen périodique (quinquennal) de santé, sous l'égide de la Caisse Nationale d'Assurance Maladie (CNAM). La quatrième visite de cette cohorte sera organisée à partir de 2011 dans le but de collecter les données à 17 ans pour permettre à tous les utilisateurs potentiels ayant un intérêt à cette visite de reprendre leurs travaux scientifiques de la cohorte. Une enquête préliminaire sous l'égide du Comité Scientifique a permis de collecter les déclarations d'intention des investigateurs.

Une étude ancillaire intitulée « Mesure d'indices fonctionnels cardiaques en IRM à l'aide de la technique GRICS » (Dr Laurent Bonnemains – CIC-IT de Nancy) sera également proposée à 76 participants à l'étude.

La technique de post-traitement des données IRM *Generalized Reconstruction by Inversion of Coupled System* (GRICS), développée par le laboratoire IADI, permet de reconstruire les images d'un objet en mouvement à partir de l'acquisition IRM de cet objet et de données caractérisant le mouvement. L'utilisation de GRICS rend également accessible, lors d'une acquisition IRM, le calcul d'indices fonctionnels cardiaques jusqu'alors réservés à l'échocardiographie (faisabilité démontrée sur 5 volontaires sains).

Objectif principal de l'étude ancillaire « Mesure d'indices fonctionnels cardiaques en IRM à l'aide de la technique GRICS (Dr Laurent Bonnemains – CIC-IT de Nancy) » :

- Comparer les indices fonctionnels cardiaques mesurés en IRM à l'aide de la technique GRICS avec les indices équivalents mesurés en échocardiographie.

Objectif secondaire de l'étude ancillaire:

- Estimer la reproductibilité inter observateur de ces indices fonctionnels.

Méthodologie, Organisation :

Dans le cadre de l'étude ancillaire, un examen d'IRM cardiaque sera proposé à 76 participants à l'étude.

2 JUSTIFICATION SCIENTIFIQUE ET DESCRIPTION GENERALE DE LA RECHERCHE :

L'Imagerie par Résonance Magnétique (IRM) est une modalité d'imagerie non invasive et non ionisante (pas d'utilisation de rayons X). Cette technique est basée sur l'utilisation d'un champ magnétique intense et d'ondes radiofréquences pour créer des images anatomiques et fonctionnelles. Cette technique est aujourd'hui considérée comme une technique d'exploration anatomique et fonctionnelle de référence. Depuis ses débuts vers les années 1985, cette technique est en permanente évolution. Un grand nombre de paramètres techniques (séquence, paramètres dans la séquence, antenne, champ magnétique 1,5 ou 3T, méthode de reconstruction, etc.) peuvent être changés pour optimiser la qualité diagnostique de l'examen.

Une des avancées majeures de la technique de post-traitement des données IRM a été récemment apportée par le laboratoire IADI (Imagerie Adaptative Diagnostique Interventionnelle - INSERM U947 - Université de Lorraine) qui a développé la technique *Generalized Reconstruction by Inversion of Coupled System* (GRICS). Cette méthode permet de reconstruire les images d'un objet en mouvement à partir de l'acquisition IRM de cet objet (donc d'images floues) et de données caractérisant le mouvement, généralement issues de capteurs (ceintures respiratoire, ECG et accéléromètres essentiellement) ou de navigateurs (utilisation d'une partie des données IRM pour détecter le mouvement).

C'est une méthode rétrospective qui opère une correction a posteriori des données IRM acquises sans contrainte a priori sur le type de mouvement.

Le contraste de phase avec haute résolution temporelle permet de calculer les indices fonctionnels habituellement accessibles uniquement en échographie par doppler tissulaire, tels que la vitesse de l'anneau ou le Myocardial Performance Index (MPI).

Ces indices fonctionnels (strain, vitesse de l'anneau, ou MPI entre autres) sont aujourd'hui bien connus et utilisés en pratique courante en échocardiographie. Ils ne sont pas calculables avec la technique IRM conventionnelle. Leur utilisation en IRM constituerait une amélioration importante de la qualité diagnostique de nos examens.

3 OBJECTIFS DE LA RECHERCHE :

3.1 OBJECTIF PRINCIPAL :

Comparer les indices fonctionnels cardiaques mesurés en IRM à l'aide de la technique GRICS avec les indices équivalents mesurés en échocardiographie Doppler.

3.2 OBJECTIF SECONDAIRE :

Evaluation de la reproductibilité inter-opérateur de l'échocardiographie Doppler et de l'IRM.

3.3 CRITERES D'EVALUATION PRINCIPAUX :

Comparaison par paires (IRM et échographiques) des indices fonctionnels cardiaques pour chaque sujet : vitesse de l'anneau tricuspide et mitral, MPI et strain myocardique pour les deux ventricules.

La liste des paramètres échographiques et IRM destinés à être comparés est fournie à titre indicatif : elle est susceptible d'être complétée en fonction de l'évolution des connaissances, à l'occasion des relectures de ces examens.

3.4 CRITERES D'EVALUATION SECONDAIRES :

Comparaison des deux mesures des indices fonctionnels cardiaques ci-dessus effectuées pour chaque méthode.

4 CONCEPTION DE LA RECHERCHE

4.1 MODALITES DE RECRUTEMENT

Les volontaires acceptant de participer au point de suivi à 17 ans de la cohorte STANISLAS, seront sollicités pour participer à cette étude ancillaire en fonction du planning de disponibilité des IRM. Le sujet en sera informé lors du contact téléphonique établi dans le cadre de l'étude principale.

Le jour de la visite programmée (si la visite a lieu un jour de disponibilité de l'IRM)

- L'investigateur informera le sujet des objectifs et du déroulement de l'étude ancillaire.
- L'investigateur remettra au volontaire la lettre d'information et le formulaire de consentement ainsi que le questionnaire de contre-indication à l'IRM.

A l’issue de l’échocardiographie réalisée dans le cadre de l’étude principale :

- Le groupe approprié sera déterminé et le volontaire confirmera son souhait de participer, en supposant que l’effectif requis ne soit pas atteint dans son groupe. Le formulaire de consentement paraphé et signé par le volontaire sera recueilli par le médecin investigateur qui le paraphe et le signera à son tour.
- Le questionnaires de contre-indication à l’IRM sera validé par un entretien avec le médecin investigateur, qui verifiera également les critères d’inclusion et de non inclusion.
- Pour les femmes en âge de procréer, en cas de doute sur une éventuelle grossesse débutante, un test urinaire de grossesse sera effectué le jour de la visite. L’investigateur s’assurera des résultats de ce test et en cas de résultat positif, elles seront sorties de l’étude. Ce test sera pris en charge par le CIC-IT.

4.2 DEROULEMENT DE L’EXAMEN IRM CARDIAQUE

L’étude se fera sur les IRM du CHU de Nancy Brabois utilisées en pratique clinique (installations de marque General Electric (GE)). Toutes ces installations sont homologuées et utilisées aussi bien en clinique qu’en recherche dans le monde entier (environ 20 000 IRM installées dans le monde).

Comme lors de l’examen IRM standard, le sujet sera préparé (retrait des effets ferromagnétiques, cartes de crédit, montre,...) puis installé par le manipulateur IRM dans la salle d'IRM.

Le sujet sera ensuite introduit dans l'aimant de l'IRM une fois le fonctionnement des différents capteurs vérifié (capteurs externes habituels, à savoir 2 ceintures pneumatiques et un capteur ECG).

L’examen IRM consistera à l’application des différentes séquences d’acquisition d’images (séquences en contraste de phase de type PC-Cine et séquence de ciné-IRM) par le manipulateur IRM.

La durée à l’intérieur de l’aimant sera en moyenne de 45 minutes. Cependant, le sujet pourra être sorti de l’imageur à n’importe quel moment de l’étude à sa demande. En effet, un système de communication permet un dialogue régulier entre le volontaire situé dans l’IRM et le manipulateur situé en dehors de la salle IRM.

Les indices fonctionnels cardiaques en IRM seront ensuite mesurés à l’aide de la technique GRICS puis comparés à leurs équivalents issus de l’échocardiographie pour chaque sujet.

Une seconde lecture des mesures échocardiographiques et IRM sera effectuée ultérieurement par un autre opérateur (différent du premier) pour permettre l'évaluation de la reproductibilité de chacune des 2 méthodes.

4.3 CALENDRIER DE L'ETUDE PRINCIPALE

Date de début d'étude : 05/07/2011
Durée théorique de recrutement pour atteindre le nombre de sujets nécessaires : 48 mois
Durée de participation pour un sujet : 1 journée
Date théorique de fin d'étude correspondant au terme de la participation de la dernière personne qui se prête à la recherche : 05/07/2015

4.4 IDENTIFICATION DE TOUTES LES DONNEES A RECUEILLIR

Les principales données à recueillir seront les suivantes :

- Sexe, Poids, Taille,
- Critères inclusion et non inclusion de l'étude ancillaire
- Données IRM (date de l'IRM, mesures des indices fonctionnels cardiaques effectués, données relatives à la machine : IRM 1,5T ou 3T et antenne utilisée)

L'ensemble des données recueillies dans le cahier d'observation doit pouvoir être confronté aux données du dossier source du sujet.
Les auto-questionnaires seront considérés comme données source. Il en sera de même pour les données brutes de l'échographie ainsi que les données brutes de l'IRM, les images et les données des capteurs, qui seront stockées dans la base de données ARCHIMED déclarée à la CNIL.

5 POPULATION DE L'ETUDE ANCILLAIRE

Cette étude pilote inclura 76 sujets (cf justification de l'effectif) sélectionnés dans l'étude principale. Ces sujets seront répartis en 2 groupes égaux de 38 sujets définis par l'absence ou la présence d'une anomalie pour au moins un des indices à l'échocardiographie Doppler afin de maximiser l'étendue des mesures et la sensibilité de l'analyse de concordance entre les 2 méthodes. Les sujets seront enrôlés consécutivement dans l'étude, en fonction des disponibilités de l'IRM, jusqu'à obtention de l'effectif requis dans chaque groupe.

Le groupe avec échocardiographie normale sera constitué de volontaires ayant des indices de fonction systolique ou diastolique tous normaux lors de l'échocardiographie de la visite d'inclusion : fraction d'éjection, vitesse du pic de l'onde S tricuspide et mitrale, rapport E/Ea, MPI et strain longitudinal global pour les deux ventricules.

L'autre groupe sera constitué de volontaires pour lesquels l'échographiste aura noté une anomalie pour au moins un des indices suivants : fraction d'éjection, vitesse du pic de l'onde S tricuspide et mitrale, rapport E/Ea, MPI et strain longitudinal global pour les deux ventricules.

Les seuils utilisés pour discriminer les deux groupes à l'échographie seront choisis par consensus entre les investigateurs au démarrage de l'étude en fonction de l'état des connaissances médicales à ce moment (état de l'art pouvant changer dans ce domaine).

5.1 CRITERES D'INCLUSION

- Obtention du consentement éclairé et écrit pour l'étude principale.
- Obtention du consentement éclairé et écrit pour l'étude ancillaire.

5.2 CRITERES DE NON-INCLUSION

- Contre-indication à l'examen IRM, en particulier stimulateur cardiaque ou défibrillateur implantable, pompe implantée, implants cochléaires, clips neurochirurgicaux, corps étrangers métalliques intra orbitaires ou encéphaliques,
- Impossibilité de réaliser l'examen IRM, en particulier claustrophobie, morphotype ne permettant pas l'accès à l'IRM (diamètre abdominal trop important),
- Les femmes en âge et en état de procréer n'ayant pas de méthode contraceptive efficace,
- les femmes enceintes.

5.3 PROCEDURE D'ARRET PREMATURE DE LA RECHERCHE OU D'EXCLUSION POUR UNE PERSONNE DE LA RECHERCHE ET PROCEDURE DE SUIVI DE LA PERSONNE

Seront exclus définitivement de l'étude ancillaire :

- Sujets revenant sur leur consentement,
- Les sujets n'ayant pas effectué l'examen IRM au complet,
- Examen IRM effectué au complet mais avec des données manquantes.
- Apparition d'un critère de non-inclusion

6 CONSERVATION ET ACCES AUX DONNEES CLINIQUES ET PARACLINIQUES

Toutes les données relatives aux participants seront anonymisées au CIC-P.
L'ensemble des données anonymisées pourra être exporté conformément aux réglementations en vigueur et utilisé par des équipes de recherche nationales et internationales.

7 EVALUATION DE LA SECURITE :

L'IRM constitue une technique d'imagerie ne présentant pas de danger pour l'homme si les règles de sécurité (essentiellement en ce qui concerne les éléments ferromagnétiques) sont parfaitement respectées.
L'étude se fera sur les IRM cliniques du CHU de Nancy, une IRM à 1,5T et une IRM à 3T (dédiée à 40% à la recherche). Toutes ces IRM sont homologuées et utilisées aussi bien en clinique qu'en recherche dans le monde entier (environ 30 000 IRM installées dans le monde). Ces machines sont régulièrement mises à jour dans le cadre d'un partenariat et d'un contrat de maintenance. Toutes les limites imposées par les normes sont respectées et toujours contrôlées automatiquement pendant les acquisitions.
Notons bien l'absence de tout acte médical invasif (pas d'injection). En cas de découverte fortuite d'une anomalie morphologique sur les images, une consultation sera proposée au volontaire avec un spécialiste du CHU ou son médecin traitant.

Les effets attendus peuvent être les désagréments habituels, mais sans gravité, liés à l'examen IRM (par exemple, céphalées, sensation de chaleur).
Les évènements indésirables seront recueillis par les investigateurs pendant toute la durée de réalisation de l'IRM. Ils seront retranscrits dans le cahier d'observation.
Les recommandations et les procédures relatives à l'IRM, rédigées afin d'assurer la sécurité des sujets et mises à jour par les sociétés savantes de Radiologie, constitueront le document de référence afin de définir le caractère attendu d'un EIG.

8 STATISTIQUES

8.1 DESCRIPTION DES METHODES STATISTIQUES PREVUES

Les différents données de cette étude ancillaire seront décrits par leurs moyennes ± écart-type, médiane [percentile à 25% - percentile à 75%], maximum et minimum. L'analyse du critère principal (concordance des méthodes GRICS et échographie-Doppler) sera effectuée sur les données moyennes calculées à partir des résultats de la double lecture, selon la méthode de Bland et Altman. L'analyse de reproductibilité des 2 techniques sera effectuée de la même manière. Ces analyses seront complétées par le calcul du coefficient de corrélation intraclasse (inter-technique et inter-opérateur) par ANOVA mixte avec le sujet comme facteur aléatoire.

8.2 Nombre prévu de personnes à inclure dans la recherche, avec sa justification statistique

Avec 60 sujets, la moyenne des différences pourra être déterminée avec une précision (demi-intervalle de confiance à 95%) < 0.26 DS, et les limites de son intervalle de confiance à 95% avec une précision < 0.10 DS. Le nombre total de sujets à inclure dans l'étude est porté à 76 afin de compenser les observations non utilisables du fait des prévisibles problèmes de mesure par l'une et/ou l'autre des 2 méthodes. Deux groupes de 38 sujets avec et sans anomalie à l'échocardiographie-doppler seront recrutés afin de d'élargir le spectre des mesures observées et de maximiser la sensibilité de l'analyse.

Centre hospitalier universitaire de Nancy - Direction de la Recherche et de l'Innovation
**Clinical trial protocol with medical device**<br>**Amendment to the STANISLAS 4th visit protocol adding the ancillary study ESCIF**
Main protocol : HÉRITABILITÉ FAMILIALE, INTERACTIONS GÈNE-GÈNE ET GÈNE-ENVIRONNEMENT DANS LE DOMAINE DES MALADIES CARDIOVASCULAIRES<br>Quatrième visite (à 17 ans) de la Cohorte STANISLAS<br><br>Ancillary study : ESCIF

Accepted by the CPP Est III the 03/02/2011

Accepted by the french governement agency (AFSSAPS) the 28/01/2011

Version N°3 (03/08/2012)

N°ID-RCB : 2010-A01469-30

Promotor :

Centre Hospitalier Universitaire de Nancy
Avenue du Maréchal de Lattre de Tassigny
54035 NANCY Cedex

Main Investigator:

Name: Pr. Faiez ZANNAD
Address: Centre d'investigation Clinique, Institut lorrain du Cœur et des Vaisseaux Louis Matthieu, 4 rue du Morvan, 54500 Vandoeuvre lès Nancy
Phone: 03 83 15 73 20
Mail: f.zannad@chu-nancy.fr

Research responsible and promotor delegate:
Direction de la Recherche et de l'Innovation
M. Olivier DE PESQUIDOUX – Directeur de la Recherche et de l'Innovation
Hôpital Saint Julien – Rue Foller
CO N°60034
54035 NANCY Cedex

SUMMARY

1 Summary of the protocole 13

2 Scientific justification and general description of the research 14

3 OBJECTIVES AND EVALUATION CRITERIA 14

3.1 Principal Objective 14

3.2 Secondary Objective 15

3.3 Principal Evaluation Criterion 15

3.4 Secondary Evaluation Criterion 15

4 METHODS 15

4.1 Inclusions 15

4.2 The MRI cardiac exam 16

4.3 Calendar of the main study 16

4.4 Data collected 16

5 POPULATION DE L'ETUDE ANCILLAIRE 16

5.1 inclusion CRITERIA 17

5.2 non-inclusion CRITERIA 17

5.3 EXCLUSION CRITERIA 17

6 Conservation ACCESS TO CLINICAL AND PARACLINICAL DATA 17

7 SECURITY EVALUATION 18

8 STATISTICS .... **Erreur ! Signet non défini.**

8.1 Statistic methods 18

8.2 SIZE OF THE POPULATION 18

1 SUMMARY OF THE PROTOCOLE

Context:

The STANISLAS cohort is a monocentric familial longitudinal cohort composed of 1006 famillies (4295 subjects) living in/near Nancy. They were included in 1993-1995 by the Centre de Médecine Préventive (CMP) of Vandoeuvre lès Nancy and underwent periodical (every 5 years) systematic health exam under the direction of the Caisse Nationale d'Assurance Maladie (CNAM). The fourth visit of this cohort will begin in 2011 in order to collect data 17 years after the inclusion and to allow all the potential users of the cohort to continue their work.

The ESCIF ancillary study, proposed by Dr Laurent Bonnemains (CIC-IT, Nancy), concerns 76 volunteers of the 4th visit of the Stanislas Cohort. Its aim is to compare cardiac functional indices acquired with MRI and with trans-thoracic echocardiography.

The IADI laboratory has developed several technics aiming at acquiring MRI images of moving organs either at high temporal resolution or with motion correction such as with the post processing technic named GRICS *Generalized Reconstruction by Inversion of Coupled System.* The use of GRICS or other technics allow us to compute from MRI acquisitions various cardiac functional indices which were up to now only used in echocardiography. At this moment, the feasibility of these technics has been shown on 5 healthy volunteers.

Principal objective of the ancillary study « Mesure d'indices fonctionnels cardiaques en IRM à l'aide de la technique GRICS (Dr Laurent Bonnemains – CIC-IT de Nancy) » :

- To compare cardiac functional indices measured with MRI using technics derived from GRICS with equivalent indices measured with echocardiography.

Secondary Objective of the ancillary study

- To assess the interobserver reproducibility of these indices.

Methodology, Organization:

For the ancillary study ESCIF, a specific MRI cardiac examination will be proposed to 76 participants of the Stanislas cohort.

2 SCIENTIFIC JUSTIFICATION AND GENERAL DESCRIPTION OF THE RESEARCH

Magnetic Resonnance Imaging (MRI) is a non-invasive technic using no radiations. It is based on a high static magnetic field coupled with radio-frecquency waves. It can produce anatomical and functionnal images. It can be considered nowadays as a reference technic for these purposes. Since the beginning of MRI around 1985, it has evolved a lot. A lot of parameters can be tuned to optimize the diagnostic capacity of the exam (type and parameters of the sequence, antenna, strength of the field, reconstruction method, …)

One of these major improvement in the field of post-treatment was brought by the IADI lab which proposed recently the GRICS technic (Generalized Reconstruction by Inversion of Coupled System).This technic allows the reconstruction of moving objects with motion correction based on external data generally coming from external captors such as belts or accelerometers or based on internal data coming from the MRI acquisition itself (such as with navigators).

The motion detection and correction is therefore retrospective and works on data acquired freely without any constraint on the type of motion.

High temporal resolution phase contrast acquisitions could allow the measurement of indices yet only accessible with tissue-doppler imaging in echocardiographysuch as the Myocardial Performance Index (MPI). These functional indices (MPI and others) are at the moment used in clinical practice in echography but not in MRI. Their use in MRI could constitute an important improvement of the diagnostic quality of our exams.

3 OBJECTIVES AND EVALUATION CRITERIA

3.1 PRINCIPAL OBJECTIVE

To compare cardiac functional indices measured with MRI using technics derived from GRICS with equivalent indices measured with echocardiography.

3.2 SECONDARY OBJECTIVE

To assess the interobserver reproducibility of these indices.

3.3 PRINCIPAL EVALUATION CRITERION

Paired comparison (MRI vs echocardiography) of cardiac functional indices for each subject: for example, velocity of tricuspid or mitral annulus, MPI and myocardial strains for both ventricles. The list of MRI and echographic parameters to be compared is indicative and will be completed when knowledge will evolved and when the exams will be processed.

3.4 SECONDARY EVALUATION CRITERION

Bland and Altman comparisons of two assessments of each parameter.

4 METHODS

4.1 INCLUSIONS

The volunteers accepting to participate to the fourth visit (at 17 years) of the STANISLAS cohort will be proposed to participate into the ESCIF ancillary study, whenever the MRI scanners will be available the day of their visit. Subjects will be informed during a previous phone call, planned by the main Stanislas study.

The day of the 4th STANISLAS visit (if the MRI scanners are available)

- The investigator will inform the volunteer of the objectives and methods of the ancillary study.
- The information letter and consent form will be presented and given to the STANISLAS volunteer, with the MRI non contra-indication questionnary.

At the end of the echocardiography (comprised into the main STANISLAS study)

- The volunteer group will be chosen (group 1 if the echography is normal, group 2 if at least one index is not normal). The volunteer will give his consent to participate into the ancillary study (if the inclusions for this group are not closed). The consent form will be signed by the volunteer and by the investigator.
- MRI non contra-indication questionary will be validated after discussion with the investigator.
- Concerning women able to become pregnant, in case there is a possibility of incoming pregnancy, a urinary pregnancy test will be performed. The investigator will check this test and the woman will be excluded of the study if the result is positive.

4.2 THE MRI CARDIAC EXAM

The exam will be performed using one of the General Electric MRI scanners of the CHU Nancy Brabois. These scanners are used every day for clinical practice and/or for research protocols. They have been homologated and more than 20000 of them are used worldwide.

As for a conventional cardiac MRI exam, the subject will be prepared and installed by the MRI radiographer. The subject will then be put inside the bore, once the different captors will have proven that they work (two pneumatic belts and one ECG captor).

The MRI protocol will be composed of differents sequences, based either on phase contrast cine MRI, or SSFP cine MRI. The duration of the examination will be around 45 minutes. However, the volunteer will be allowed to leave the bore whenever he will request. Indeed, a communication system will allow a periodic conversation between the volunteer and the radiographer. The functional cardiac indices measured in MRI with technics based on the GRICS method will be compared to their equivalent assessed during the echocardiographic exam a few minutes before.

A second assessment of these indices will be performed later to allow the assessment of inter-observer reproducibility of both methods.

4.3 CALENDAR OF THE MAIN STUDY

Beginning of the main study: 05/07/2011

Theoretical delay necessary to include everybody : 48 month

Participation of each volunteer : 1 day

Theoretical end of the study : 05/07/2015

4.4 DATA COLLECTED

The main data collected will be:

- Sex, weight, height
- Inclusion criteria and non inclusion criteria
- MRI and echocardiographic data

The echocardiographic and MRI raw data an images as well as the captors data will be stored in a ARCHIMED database declared to the french governement agency (CNIL).

5 POPULATION DE L'ETUDE ANCILLAIRE

This study will comprise 76 subjects (cf population size justification) consecutively selected from the main STANISLAS cohort. This subjects will be included into two groups of 38

subjects. Group one subjects will have a complete echocardiographic exam considered normal (ejection fraction, maximal S wave at the tricuspid and mitral annulus, ratio E/Ea, MPI, longitudinal strain for both ventricles), whereas group 2 subjects will have at least one abnormally in this exam. This design was chose to obtain widely spread values for the tested indices to maximize the sensitivity of the concordance study.

Subjects will be enrolled in the study consecutively according to the availability of the MRI scanners, until the size of each group will be reached.

The thresholds chosen to discriminate group 1 from group 2 will be chosen by consensus at the beginning of the study (the state of the art may change rapidly in this field of knowledge).

5.1 INCLUSION CRITERIA

- Contentment for the main study: Stanislas 4th visit.
- Contentment for the ancillary study.

5.2 NON-INCLUSION CRITERIA

- Contra-indication to MRI, notably pacemaker or defibrillator, pumps, cochlear implants, neurosurgical clips, intra-ocular metallic elements, …
- Impossibility to perform MRI, notably because of claustrophobia, obesity…
- Pregnancy

5.3 EXCLUSION CRITERIA

- when volonteers have withdrawn their consent
- when the MRI exam was not complete,
- if a non-inclusion criterion appears

6 CONSERVATION ACCESS TO CLINICAL AND PARACLINICAL DATA

Every data concerning volunteers of the study will be anonymized by the CIC-P.

It will be possible after anonymization to share these data in accordance with existing regulations concerning national or international research teams.

7 SECURITY EVALUATION

MRI is an imaging technic presenting no risk for human being when security rules (especially those concerning metallic items) are respected. The ESCIF study will be performed on two MRI scanners (1.5 and 3T) within the CHU of Nancy-Brabois. These MRI scanners are homologated and used world widely for clinical or research purpose (more than 20000 scanners in the world). These machines are regularly updated with a support maintenance contract. All the limits imposed by norms will be respected and automatically controlled during the acquisitions. There will be no invasive act (no gadolinium injection). In case of unexpected discovery of any anatomical anomaly, a medical consultation will be proposed to the volunteer with a specialist physician of the CHU or with the usual GP.

The expected effects of the MRI can comprise: headache, heat sensation…
The adverse events will be noted by the investigators during the duration of the examination. They will be noted on the CRF. Documents written by national and international societies of Radiology concerning MRI will be used to define the expected or unexpected character of an adverse event.

8 STATISTICS

8.1 STATISTIC METHODS

Data will be described using mean ± standard-deviation, median [25% percentile - 75% percentile], maximum and minimum. The main objective analysis will be performed with a Bland-Altman comparison of each parameter assessed both in MRI and in echocardiography. The reproducibility of each method will also be assessed with Bland-Altman method and will be completed by the computation of ICC (inter technic and inter observer) with a mixed ANOVA using the volunteer as random factor.

8.2 SIZE OF THE POPULATION

With 60 volunteers, the mean of the differences could be assessed with a precision (half 95% interval) < 0.26 SD, and the limits of its 95% confidence interval with a precision < 0.10 SD.

The total number of subject to include was augmented to 76 to take into account the possible missing data due to unavoidable events during MRI acquisition or discovered during echocardiographic post treatment.

Two groups of 38 subjects with/without echocardiographic anomaly will be recruited to maximize the opportunity to obtain widely spread parameters.
