# Supplementary material for: Accuracy of subject-specific prediction of end-systolic time in MRI across a range of RR intervals
Source: PLoS One. 2017 Jun 9;12(6):e0179011. doi: 10.1371/journal.pone.0179011 (PMC5466307; doi:10.1371/journal.pone.0179011)
Supplement: S1 Table — Discarded subjects (under the quality criterion) are marked with an ‘x’. (DOCX) [file pone.0179011.s003.docx]

Supporting Table S1. Sex, age, slope and intercept and quality metric of the Patient-Adapted Cardiac Model for each subject. Discarded subjects (under the quality criterion) are marked with an ‘x’

| Subject | Sex | Age | Slope [s/bpm] (CI) | Intercept [s] (CI) | SD [s] |
| --- | --- | --- | --- | --- | --- |
| 1 | M | 34 | -0.0009 (+/- 0.0002) | +0.372 (+/- 0.015) | 0.006 |
| 2 | F | 31 | -0.0006 (+/- 0.0002) | +0.388 (+/- 0.016) | 0.004 |
| 3 | M | 59 | -0.0015 (+/- 0.0005) | +0.457 (+/- 0.031) | 0.004 |
| 4 | M | 60 | -0.0022 (+/- 0.0015) | +0.465 (+/- 0.070) | 0.008 |
| 5 | M | 40 | -0.0004 (+/- 0.0003) | +0.367 (+/- 0.020) | 0.006 |
| 6 x | F | 39 | NA | NA | 0.013 |
| 7 | F | 63 | -0.0007 (+/- 0.0003) | +0.379 (+/- 0.025) | 0.004 |
| 8 | M | 64 | -0.0016 (+/- 0.0005) | +0.445 (+/- 0.032) | 0.005 |
| 9 | F | 66 | -0.0007 (+/- 0.0005) | +0.423 (+/- 0.027) | 0.006 |
| 10 | M | 69 | -0.0009 (+/- 0.0007) | +0.411 (+/- 0.041) | 0.006 |
| 11 | F | 60 | -0.0033 (+/- 0.0006) | +0.553 (+/- 0.040) | 0.004 |
| 12 | F | 56 | -0.0007 (+/- 0.0005) | +0.383 (+/- 0.034) | 0.004 |
| 13 | F | 52 | -0.0010 (+/- 0.0003) | +0.398 (+/- 0.021) | 0.008 |
| 14 | F | 39 | -0.0015 (+/- 0.0002) | +0.461 (+/- 0.012) | 0.006 |
| 15 | F | 43 | -0.0007 (+/- 0.0010) | +0.392 (+/- 0.067) | 0.006 |
| 16 | F | 57 | -0.0010 (+/- 0.0009) | +0.431 (+/- 0.057) | 0.006 |
| 17 x | M | 56 | NA | NA | 0.018 |
| 18 | F | 54 | -0.0015 (+/- 0.0005) | +0.453 (+/- 0.039) | 0.005 |
| 19 | F | 35 | -0.0009 (+/- 0.0001) | +0.427 (+/- 0.009) | 0.003 |
| 20 | M | 58 | -0.0004 (+/- 0.0002) | +0.355 (+/- 0.017) | 0.006 |
| 21 | F | 57 | -0.0008 (+/- 0.0005) | +0.366 (+/- 0.040) | 0.003 |
| 22 | F | 37 | -0.0003 (+/- 0.0004) | +0.363 (+/- 0.028) | 0.005 |
| 23 | F | 35 | -0.0010 (+/- 0.0002) | +0.371 (+/- 0.017) | 0.002 |
| 24 x | M | 37 | NA | NA | 0.011 |
| 25 | M | 56 | -0.0021 (+/- 0.0010) | +0.494 (+/- 0.066) | 0.005 |
| 26 | M | 58 | -0.0010 (+/- 0.0005) | +0.405 (+/- 0.032) | 0.007 |
| 27 | F | 56 | -0.0018 (+/- 0.0005) | +0.444 (+/- 0.028) | 0.003 |
| 28 | M | 64 | +0.0004 (+/- 0.0010) | +0.307 (+/- 0.055) | 0.006 |
| 29 | F | 64 | -0.0008 (+/- 0.0007) | +0.411 (+/- 0.043) | 0.007 |
| 30 | F | 57 | -0.0008 (+/- 0.0004) | +0.371 (+/- 0.028) | 0.005 |
| 31 | M | 61 | -0.0011 (+/- 0.0003) | +0.392 (+/- 0.022) | 0.007 |
| 32 | F | 37 | -0.0005 (+/- 0.0002) | +0.389 (+/- 0.011) | 0.004 |
| 33 | M | 53 | -0.0006 (+/- 0.0005) | +0.412 (+/- 0.028) | 0.005 |
| 34 x | M | 60 | NA | NA | 0.029 |
| 35 | M | 57 | -0.0010 (+/- 0.0004) | +0.434 (+/- 0.025) | 0.005 |

*All regression coefficients are significant to P < 0.005*

*SD = sample standard deviation from regression in seconds
CI = confidence interval at 95% level*
